# Targeting of the non-mutated tumor antigen HER2/neu to mature dendritic cells induces an integrated immune response that protects against breast cancer in mice

**DOI:** 10.1186/bcr3135

**Published:** 2012-03-07

**Authors:** Bei Wang, Neeha Zaidi, Li-Zhen He, Li Zhang, Janelle MY Kuroiwa, Tibor Keler, Ralph M Steinman

**Affiliations:** 1 Laboratory of Cellular Physiology and Immunology and Chris Browne Center of Immunology and Immune Disease, The Rockefeller University, 1230 York Ave, New York, NY 10065, USA; 2 Celldex Therapeutics, Inc., 222 Cameron Drive, Suite 400, Phillipsburg, NJ 08865, USA

## Abstract

**Introduction:**

Given their relative simplicity of manufacture and ability to be injected repeatedly, vaccines in a protein format are attractive for breast and other cancers. However, soluble human epidermal growth factor receptor (HER2)/neu protein as a vaccine has not been immunogenic. When protein is directly targeted to antigen uptake receptors, such as DEC205 (DEC), efficient processing and presentation of antigen take place. The aim of this study was to determine the immunogenicity of a HER2 protein vaccine that directly targets to DEC^+ ^dendritic cells (DCs) in a mouse breast cancer model.

**Methods:**

We genetically engineered the HER2 extracellular domain into a monoclonal antibody specific for DEC (DEC-HER2). Mice of various genetic backgrounds were immunized with DEC-HER2 in combination with DC maturation stimuli (poly IC ± CD40 Ab). Vaccine-induced T cell immunity was determined by analyzing the ability of CD4^+^/CD8^+ ^T cell to produce interferon (IFN)-gamma and proliferate upon antigen rechallenge. Sera were assessed for the presence of antigen specific antibody (Ab). For vaccine efficacy, FVB/N mice were immunized with DEC-HER2 in combination with poly IC and protection against neu-expressing mammary tumors was assessed. Protection mechanisms and tumor-specific T cell responses were also evaluated.

**Results:**

We demonstrate that DEC-HER2 fusion mAb, but not Ctrl Ig-HER2, elicits strong, broad and multifunctional CD4^+ ^T cell immunity, CD8^+ ^T cell responses, and humoral immunity specific for HER2 antigen. Cross-reactivity to rat neu protein was also observed. Importantly, mice xeno-primed with DEC-HER2 were protected from a neu-expressing mammary tumor challenge. Both CD4^+ ^and CD8^+ ^T cells mediated the tumor protection. Robust anti-tumor T cell immunity was detected in tumor protected mice.

**Conclusions:**

Immunization of mice with HER2 protein vaccine targeting DEC^+ ^DCs *in vivo *induced high levels of T- and B-cell immunity. Non-targeted HER2 protein was poorly immunogenic for CD4^+ ^and CD8^+ ^T cells. This vaccination approach provided long-term survival benefit for mice challenged with neu-expressing tumor following as little as 2.7 μg of HER2 protein incorporated in the vaccine. Vaccine-induced CD4^+ ^and CD8^+ ^T cells were both essential for tumor protection. This immunization strategy demonstrates great potential towards the development of vaccines for breast cancer patients.

## Introduction

Despite recent diagnostic and therapeutic advances, breast cancer remains the second leading cause of cancer mortality in females in affluent countries. Targeted therapy for breast cancer has focused on receptor tyrosine kinases of the epidermal growth factor receptor (EGFR and ErbB) family, which provide critical checkpoints of cell fate decisions [1,2]. Aberrations in some members of this gene family rank among the most frequent oncogenic insults in breast cancer. The *HER2/neu *proto-oncogene encodes a tyrosine kinase growth factor receptor (p185) of the ErbB family. It is overexpressed in about 20% to 40% of invasive breast carcinomas and in approximately 70% of *in situ *ductal carcinomas. *HER2/neu *overexpression usually is associated with a poor clinical prognosis [3,4].

HER2/neu has been an attractive target for another distinct type of targeted therapy: immune therapy. Although HER2/neu is expressed by malignant cells as a non-mutated self-antigen, immune tolerance is not absolute. Both HER2/neu-specific T-cell and antibody (Ab) responses have been detected in patients with HER2/neu-expressing cancers [5-9]. Additionally, HER2-specific cytolytic T-lymphocyte response has been generated *in vitro *with T cells from patients with HER2-expressing tumors [6,10-12].

Given their relative simplicity of manufacture and ability to be injected repeatedly, vaccines in a protein format are attractive for breast and other cancers. However, soluble HER2/neu protein as a vaccine has not been immunogenic and usually has failed to confer protection against HER2/neu-expressing tumors [13-15]. Anti-tumor immunity can be enhanced when HER2 extracellular domain is fused to cytokines or combined with Abs fused to cytokines [15]. Other efforts to improve immunogenicity include mannosylation of the HER2 protein by producing the recombinant protein in yeast [16]. On the other hand, when antigen is directly targeted to antigen uptake receptors, efficient processing and presentation take place. HER2/neu protein has been incorporated into different vaccine platforms that directly target to antigen-presenting cells (APCs). Recently, several receptors, including B7-1/2 [17,18], CD11c [19], CD40 [20], mannose [21], and Fcγ receptors [22], have been tested for the delivery of HER2 antigen. Together, these studies suggest that, compared with non-targeted vaccinations, targeting HER2 to receptors expressed on APCs can improve HER2-specific T-cell responses and anti-tumor immunity against HER2-expressing tumor challenge in mouse models.

One of the dendritic cell (DC)-specific receptors that have not been explored for HER2 vaccination is the DEC-205 ('DEC', CD205) receptor, a type I C-type lectin [23]. Expression of DEC in mice is abundant on CD8α^+ ^DCs, which have a superior capacity of cross-presentation [24,25]. Although other receptors on DCs can be targeted [26,27], DEC is the only receptor that has been visualized so far on the numerous DCs within the T-cell areas of human lymphoid organs [28]. Targeting the DEC receptor leads to efficient endocytosis of antigens into endocytic vesicles containing major histocompatibility complex (MHC) class II molecules. This results in antigen uptake and T-cell stimulation that are hundred-fold more efficient than fluid-phase or solute pinocytosis [29-31]. Delivery of antigen to DEC^+^CD8α^+ ^DCs *in vivo *improves cross-presentation to CD8^+ ^T cells [31,32]. Increased antigen delivery efficiency through DEC significantly reduces the amount of protein required for the induction of T-cell immunity. Vaccine-induced T cells have cancer-resisting features, such as combined CD4^+ ^and CD8^+ ^T-cell immunity, production of T helper 1 (Th1)-type cytokines, and the ability to proliferate upon antigen re-challenge.

Previous studies have shown that ligation of DEC receptor by targeting Ab conjugated to antigen does not mature DCs but induces tolerance [30,33]. To overcome immune tolerance mediated by steady-state DCs, DC maturation adjuvants need to be included in the vaccine. Examples of potent adjuvants are synthetic double-stranded RNA, polyinosinic/polycytidylic acid (poly IC), and its more RNase-resistant analog stabilized with poly-L-lysine (poly ICLC). Both preclinical and clinical studies demonstrate that poly IC and poly ICLC are superior adjuvants for induction of potent T-cell immunity. Longhi and colleagues [34] showed that, compared with other Toll-like receptor (TLR) agonists, the TLR3 ligands poly IC and poly ICLC stand out as the most potent adjuvants for T-cell immunity when combined with DEC-gag monoclonal antibody (mAb) immunization in a mouse model. A recent clinical study by Caskey and colleagues [35] demonstrated that poly ICLC can be a reliable and authentic viral mimic for inducing innate immune response and for use as a vaccine adjuvant in humans. Poly IC is under clinical investigation in combination with a DEC-targeted HIV protein vaccine in our lab (Caskey M et al unpublished results).

The aim of this study was to determine the immunogenicity of HER2 protein vaccine that targeted to DEC^+ ^DCs in a preclinical mouse breast cancer model. To deliver HER2 protein to DEC^+ ^DCs *in situ*, we genetically engineered the HER2 extracellular domain into mAbs specific for DEC and tested the immunogenicity of this fusion mAb in mice in combination with DC maturation stimuli. For the tumor vaccine study, we xeno-primed mice with HER2 protein followed by a neu-expressing tumor challenge.

## Materials and methods

### Mice

Animal experiments were designed to fulfill the ethical and scientific principles provided by the Institutional Animal Care and Use Committee of The Rockefeller University (New York, NY, USA) (approved protocol 08117). Mice were maintained under specific pathogen-free conditions and were 6 to 8 weeks of age. C57BL/6, BALB/c, FVB/N, and HLA-A2.1 transgenic mice in the C57BL/6 background - C57BL/6-Tg(HLA-A2.1)1Enge/J - were purchased from The Jackson Laboratory (Bar Harbor, ME, USA). DEC^-/- ^mice were generated and provided by Michel Nussenzweig (The Rockefeller University) and are available from The Jackson Laboratory. At least three mice per group were used in immunization experiments.

### Cell lines

The neu-expressing mammary tumor cell line NT2.5 was derived from a spontaneous mammary tumor in female *neu*-N mice (FVB/N background). The cell line was established and kindly provided by Elizabeth M Jaffee (Johns Hopkins University School of Medicine, Baltimore, MD, USA). NT2.5 tumor cells were grown in a previously defined breast media, which consisted of RPMI (Gibco, now part of Invitrogen Corporation, Carlsbad, CA, USA) with 20% fetal bovine serum, 1% L-glutamine, 1% non-essential amino acids, 1% Na pyruvate, 0.5% penicillin/streptomycin, 0.02% gentamicin (Invitrogen Corporation), and 0.2% insulin (Sigma-Aldrich, St. Louis, MO, USA). Cells were maintained at 37°C in 5% CO_2_. The HER2 stably transfected tumor cell line, E0771/E2, was generously provided by Wei-Zen Wei (Karmanos Cancer Institute, Wayne State University, Detroit, MI, USA). Anti-CD4 (clone GK1.5) and anti-CD8 (clone 2.43) hybridoma cells were obtained from the American Type Culture Collection (Manassas, VA, USA) and maintained in accordance with its protocols.

### Construction and production of fusion monoclonal antibody

DNA coding HER2 extracellular domain (amino acid 22-653) was cloned in frame into the COOH terminus of anti-DEC (DEC-HER2) or control IgG heavy chain (Ctrl Ig-HER2) as described previously [30]. Fusion mAb was expressed by transient transfection in 293T cells and purified on protein G columns (GE Healthcare Bio-Sciences Corp., Piscataway, NJ, USA). Purified mAb was characterized by SDS-PAGE and Western blot by using anti-mouse IgG- horseradish peroxidase (IgG-HRP) (SouthernBiotech, Birmingham, AL, USA) or anti-HER2 mAb (clone 42; BD Transduction Laboratories, San Jose, CA, USA). Specific binding of the fusion mAb was verified by using Chinese hamster ovary (CHO) cells stably transfected with mouse DEC receptor. Binding was detected by flow cytometry by using phycoerythrin (PE)-conjugated goat anti-mouse IgG mAb (Jackson ImmunoResearch Laboratories, Inc., West Grove, PA, USA) and Alexa Fluor 488-conjugated mouse anti-HER2 mAb (clone 24D2; BioLegend, San Diego, CA, USA). All Abs had less than 0.125 endotoxin units per milligram in a Limulus Amebocyte Lysate assay (QCL-1000; BioWhittaker, Walkersville, MD, USA).

### Peptides

Overlapping (staggered by four amino acids) 15-mer peptides covering the HER2 and neu extracellular domain and HIV gag p24 protein were synthesized by Henry Zebroski in the Proteomics Resource Center of The Rockefeller University. The use of peptides overcomes, in large part, the need for antigen processing by APCs during the immune assays. The 161- and 147-member HER2 and neu peptide libraries were divided into seven and six pools, respectively.

### Immunization

Mice were immunized intraperitoneally with 5 μg of DEC-HER2 or Ctrl Ig-HER2 fusion mAb in combination with 50 μg of poly IC (polyinosinic/polycytidylic acid) (InvivoGen, San Diego, CA, USA). When indicated, a combination of 50 μg of poly IC and 25 μg of agonistic anti-CD40 mAb (clone 1C10) was used to mature DCs.

### Intracellular cytokine staining

Bulk splenocytes were stimulated with specific peptide pools (2 μg/mL or indicated concentration) or medium alone in the presence of a co-stimulatory anti-CD28 mAb (clone 37.51) for 6 hours. Brefeldin A (10 μg/mL) (Sigma-Aldrich) was added for the last 5 hours to accumulate intracellular cytokines. Anti-CD28 mAb was used only in a 6-hour intracellular cytokine staining assay but not in other T-cell immune assays, including enzyme-linked immunosorbent assay (ELISA), enzyme-linked immunosorbent spot (ELISPOT), and CFSE (5,6-carboxy fluorescein diacetate succinimidyl ester) dilution assays. For functional avidity analysis, graded doses (10 to 0.0016 μg/mL) of peptides were used to re-stimulate splenocytes. After stimulation, cells were washed and then incubated with anti-CD16/CD23 mAb (clone 2.4G2) to block Fcγ receptor for 15 minutes at 4°C. Cells were stained with Live/Dead Fixable Aqua vitality dye (Invitrogen Corporation), fluorescein isothiocyanate-conjugated anti-CD4 (clone RM4-5), PerCP-Cy5.5-conjugated anti-CD8 (clone 53-6.7), and Pacific blue-conjugated anti-CD3 (clone 17A2) (eBioscience, San Diego, CA, USA) for 20 minutes at 4°C. Cells were fixed, permeabilized (Cytofix/Cytoperm Plus; BD Biosciences, San Jose, CA, USA), and stained with allophycocyanin-conjugated anti-interferon-gamma (anti-IFNγ), PE-conjugated anti-IL-2, and PECy7-conjugated anti-tumor necrosis factor-alpha (anti-TNF-α) mAbs for 20 minutes at 4°C (BD Biosciences) and resuspended in stabilizing fixative (BD Biosciences). Data were collected by using a BD LSR II flow cytometer (BD Biosciences) and analyzed with FlowJo software (Tree Star, Inc., Ashland, OR, USA).

### CFSE dilution assay

A CFSE dilution assay was used to assess the proliferative capacity of T cells. Bulk splenocytes (2 × 10^7 ^cells/mL) were labeled with 2.5 μM CFSE (Invitrogen Corporation) in a 37°C water bath for 10 minutes. CFSE-labeled T cells were re-stimulated with pools of peptide (0.2 μg/mL) for 4 days, often in combination with intracellular cytokine staining of cells re-stimulated for the last 6 hours of culture.

### Mouse interferon-gamma enzyme-linked immunosorbent spot

Multi-screen-HA MAHA 54510 (Millipore, Billerica, MA, USA) plates were coated with 10 μg/mL of purified rat anti-mouse-IFNγ mAb (clone R46A2; BD Biosciences) in phosphate-buffered saline (PBS) overnight at 4°C. Plates were washed and blocked with PBS/1% bovine serum albumin (BSA) for 1 hour at 37°C. Magnetic-activated cell sorting (MACS)-purified CD8^+ ^or CD4^+ ^T cells (3 × 10^5^) were cultured for 2 days with 1 × 10^5 ^purified CD11c^+ ^spleen DCs pulsed with the peptide mix (1 μg/mL) or NT2.5 tumor lysate (10 μg/mL). Biotin-conjugated rat anti-mouse-IFNγ mAb (clone XMG 1.2, 2 μg/mL; BD Biosciences) was used as the detection Ab. After 2-hour incubation with detection Ab, spots were visualized with a Vectastain ABC kit (Vector Laboratories, Inc., Burlingame, CA, USA), followed by diaminobenzidine as the substrate (Invitrogen Corporation). Spots were counted in an ELISPOT reader (Autoimmun Diagnostika GmbH, Straβberg, Germany).

### Mouse cytokine enzyme-linked immunosorbent assay

Splenic CD4^+ ^and CD11c^+ ^cells were purified by MACS. CD4^+ ^cells (3 × 10^5^) were incubated with 1 × 10^5 ^CD11c^+ ^cells with peptide mix (2 μg/mL) in 96-well U-bottomed plates for 48 hours. Concentrations of IFNγ, IL-4, IL-10, and IL-17 in supernatant were measured by Ready-Set-Go! ELISA sets (eBioscience).

### Enzyme-linked immunosorbent assay for anti-HER2/neu antibodies

To detect HER2-specific Ab response, we produced FLAG-HER2 soluble protein by transient transfection of 293T cells and purification with anti-FLAG affinity gel (Sigma-Aldrich). The quality of FLAG-HER2 protein was verified by SDS-PAGE gel under non-reducing conditions (Figure S1 of Additional file 1). We coated high-binding ELISA plates (Nunc; Thermo Fisher Scientific Inc., Rochester, NY, USA) with 500 ng/mL (50 ng/well) of FLAG-HER2 overnight at 4°C. Plates were washed with PBS/0.1% Tween-20 and blocked with PBS/0.1% Tween 20/5% BSA for 1 hour at 37°C. Serial dilutions of serum were added to the plates and incubated for 1 hour at 37°C. Secondary goat anti-mouse IgG-specific Abs conjugated with HRP (SouthernBiotech) were added and visualized with tetramethylbenzidine (eBioscience) at room temperature for 5 to 10 minutes. To determine the IgG isotype, anti-mouse IgG1 or IgG2a Abs were used. The reported titers represent the highest dilution of sample showing an OD_450 _(optical density at 450 nm) of higher than 0.1. The data were presented as the log_10 _Ab titer. To determine whether serum IgG can bind to HER2/neu-expressing tumor cells, serial diluted serum was incubated with E0771/E2 (HER2^+^) or NT2.5 (neu^+^) tumor cells for 15 minutes at 4°C. Anti-mouse IgG-PE mAbs were used to detect the binding of serum IgG to tumor cells. Data were acquired by using a BD LSR II flow cytometer.

### Tumor protection

FVB/N mice were immunized intraperitoneally with 5 μg of DEC-HER2 or Ctrl Ig-HER2 mAb together with 50 μg of poly IC on days 0 and 28. Poly IC alone (50 μg) was injected as a negative control. Ten days after the boost immunization, mice were inoculated subcutaneously with 1 × 10^6 ^NT2.5 tumor cells in the shaved right flank. Tumor size was measured three times every week by using a caliper. Tumor volumes were estimated according to the formula: length × (width)^2 ^× 0.5. For survival analysis, tumor sizes of at least 500 mm^3 ^were defined as the experimental endpoint. For Ab depletion, 200 μg of CD4 or CD8 mAbs or both were given to mice intraperitoneally after boost immunization 9, 6, and 3 days before tumor challenge. Isotype control rat IgG was given as a negative control. Efficiency of depletion was confirmed by fluorescence-activated cell sorting (FACS) analysis of peripheral blood cells.

### Statistical analysis

All analysis was performed by using Prism 4.0 GraphPad software (GraphPad Software, Inc., San Diego, CA, USA). A two-sided Student *t *test (between two groups or conditions) was applied to compare statistical significance between peptide-specific responses and treatment groups of immunized mice. Survival studies were analyzed by Kaplan-Meier survival curves and log-rank test. Results were considered statistically significant when the *P *value was less than 0.05.

## Results

### HER2 extracellular domain can be introduced into a functional DEC antibody

**Figure 1:**
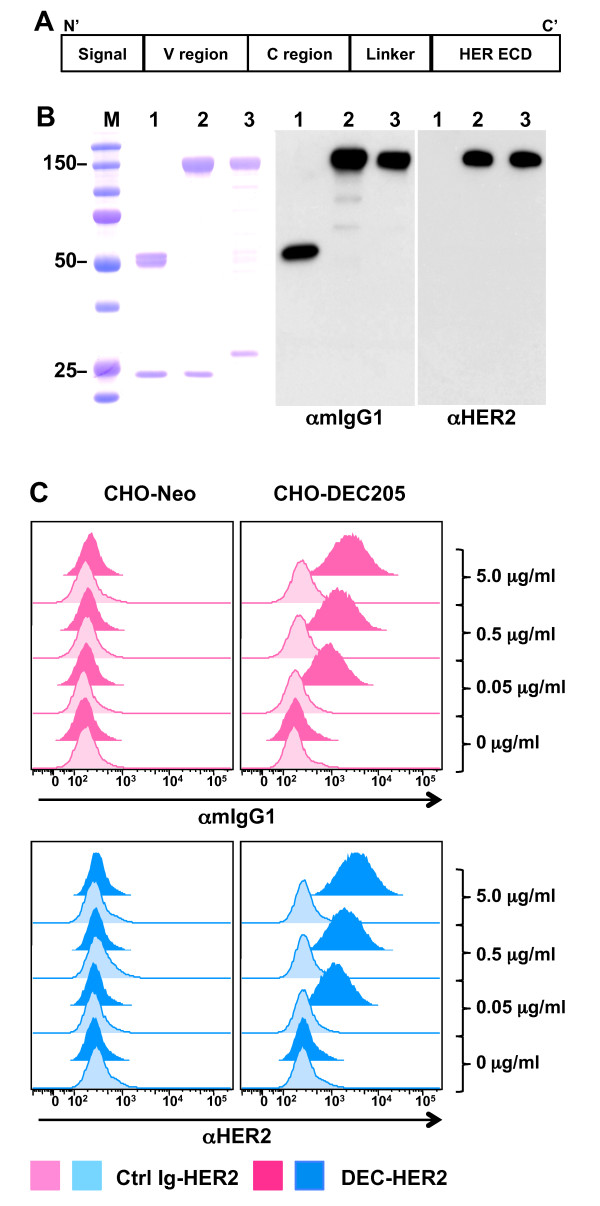
**Characterization of HER2 fusion monoclonal antibody (mAb)**. **(A) **Structure of HER2 fusion mAb. C', carboxyl-terminus; C region, constant region; ECD, extracellular domain (amino acid 22-653); N', amino-terminus; V region, variable region. **(B) **Fusion mAb (DEC-HER2 or Ctrl Ig-HER2) was produced by transient transfection of 293T cells with the appropriate vector and purified on a protein G column. Imperial protein staining (left panel) and Western blotting of the fusion mAb are shown, and the anti-mouse IgG1-HRP (αmIgG) and anti-HER2-Biotin/SAv-HRP (αHER2) antibodies are indicated below the figures. Lane 1: empty DEC mAb; lane 2: DEC-HER2 mAb; lane 3: Ctrl Ig-HER2 mAb. M, molecular weight standards (in kilodaltons). **(C) **Fluorescence-activated cell sorting staining data show the binding capacity of graded doses (0, 0.05, 0.5, and 5 μg/mL) of the indicated fusion mAb to the DEC or neomycin (Neo) stably transfected CHO cells. Binding was detected by fluorescence-labeled secondary antibody specific for mouse IgG1 or HER2 antigen. CHO, Chinese hamster ovary; HER, human epidermal growth factor receptor; Ig, immunoglobulin.

To deliver HER2 protein to mouse DCs directly *in vivo*, we cloned the extracellular domain of HER2 (amino acid 22-653) in frame into the heavy chain of the anti-mouse DEC mAb (DEC-HER2) (Figure 1A). The heavy chain of an isotype-matched non-reactive control IgG was also engineered as a non-targeting control (Ctrl Ig-HER2). The fusion mAbs were composed of a 140- to approximately 150-kDa heavy chain, consistent with a predicted mass of 90 kDa for the HER2 extracellular domain fused to the approximately 50-kDa heavy chain of unconjugated mouse IgG1 (Figure 1B). To verify whether the fusion mAb bound to the mouse DEC receptor, a stable CHO cell transfectant, expressing mouse DEC on the surface, was stained with the indicated concentration of mAb. As analyzed by FACS, DEC-HER2 mAb, but not Ctrl Ig-HER2 mAb, demonstrated proper DEC-binding activity (Figure 1C).

### DEC targeting of HER2 induces strong and broad HER2-specific CD4^+ ^T cells

**Figure 2:**
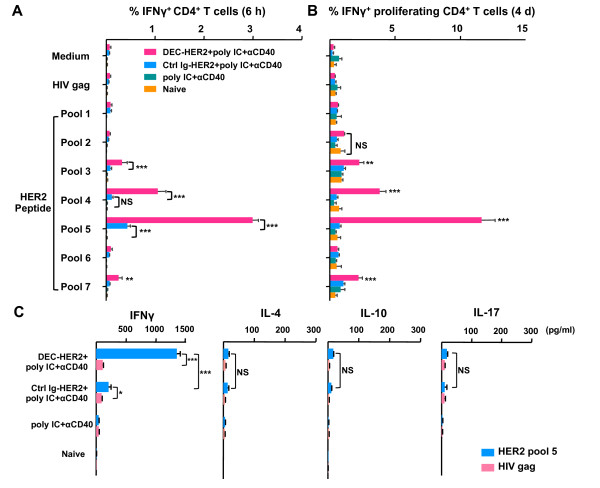
**A single dose of DEC-HER2 fusion monoclonal antibody (mAb) vaccine immunizes HER2-specific CD4^+ ^T cells *in vivo***. **(A) **Groups of C57BL/6 mice were vaccinated intraperitoneally with 5 μg of DEC-HER2 or Ctrl Ig-HER2 in combination with poly IC (50 μg) and anti-CD40 antibody (25 μg). Additional groups received adjuvants alone or were left untreated. Two weeks after immunization, splenocytes were re-stimulated without added peptides (medium alone) or with 2 μg/mL HER2 peptide pools 1 to 7 or irrelevant HIV gag peptides. Intracellular cytokine staining was determined by fluorescence-activated cell sorting after 6 hours of *in vitro *stimulation. The percentage of IFNγ^+^CD4^+ ^T cells is shown as mean ± standard error with three or four mice per group. The results of one of three independent experiments are shown. **(B) **CFSE dilution assay. Mice were immunized as in (A), and bulk splenocytes were labeled with 2.5 μM CFSE and re-stimulated with 0.2 μg of HER2 peptide pool 1-7 or HIV gag peptide mix or medium alone for 4 days. T-cell proliferation was analyzed in combination with intracellular cytokine staining of the cells re-stimulated for the last 6 hours. Percentages of proliferating (CFSE^low^) and IFNγ^+^CD4^+ ^T cells are shown with three or four mice per group. The results of one of two independent experiments are shown. **(C) **Th1/Th2/Th17 cytokine enzyme-linked immunosorbent assay (ELISA). Mice were immunized as in (A). Two weeks after the boost, splenic CD4^+ ^T cells and CD11c^+ ^cells were isolated. CD4^+ ^cells (3 × 10^5^) were incubated with 1 × 10^5 ^CD11c^+ ^cells in 96-well U-bottomed plates in the presence of 2 μg/mL HER2 peptide pool 5 or HIV gag peptides for 48 hours. Concentrations of IFNγ/IL-4/IL-10/IL-17 in culture supernatant were measured by ELISA. The results of one of two independent experiments (*n *= 4 mice per group) are shown. *P < 0.05, **P < 0.01, ***P < 0.001. CFSE, 5,6-carboxy fluorescein diacetate succinimidyl ester; HER, human epidermal growth factor receptor; IFNγ, interferon-gamma; Ig, immunoglobulin; IL, interleukin; NS, not statistically significant; poly IC, polyinosinic/polycytidylic acid; Th, T helper.

To determine the immunogenicity of the DEC-HER2 fusion mAb, we immunized C57BL/6 (H-2^b^) mice with DEC-HER2 or Ctrl Ig-HER2 intraperitoneally at a dose of 5 μg (equivalent to 14 pmol or 2.7 μg of HER2 protein). Poly IC (50 μg) and agonistic anti-CD40 mAb (25 μg) were used to mature DCs. In C57BL/6 mice, CD4^+ ^T cells responded strongly to epitopes present in HER2 peptide pools 4 and 5 (Figure 2A). We also found a weak response against HER2 epitopes in pools 3 and 7 (Figure 2A). When CD28 mAb was removed from culture, T-cell responses were reduced but remained significantly higher than background signals (Figure S2A of Additional file 2). The T-cell response was specific to HER2 since there was no reactivity to HIV gag peptides. Although Ctrl Ig-HER2 vaccination induced a significant CD4^+ ^T-cell response against peptide pool 5, its magnitude was significantly weaker than that induced by DEC-HER2 (Figure 2A). The breadth of the response was limited to pool 5. We did not observe significant CD4^+ ^T-cell responses against other pools.

In addition to determining effector T-cell pool size, we determined the proliferative capacity of vaccine-induced CD4^+ ^T cells upon antigen re-stimulation by CFSE dilution assays. Immunized T cells were stimulated with HER2 or control peptides *in vitro *for 4 days. When recalled with HER2 peptide pools for the last 6 hours, the proliferating CD4^+ ^T cells produced IFNγ specifically against HER2 (Figure 2B).

To determine whether vaccine-induced CD4^+ ^T cells are multi-functional (as defined by the combination of IFNγ, IL-2, and TNFα production at the single-cell level [36]), we measured the relative proportion of potential combinations of cytokines as depicted by pie charts and shown in Figure S3 (left panel) of Additional file 3. The mean percentage of CD4^+ ^T cells producing IFNγ/IL-2/TNFα was approximately 57%. Thus, DEC-HER2 vaccination elicited multi-functional CD4^+ ^T-cell responses.

To determine the types of Th responses induced by vaccination, we measured the production of the Th1/Th2/Th17 cytokines (IFNγ, IL-4, IL-10, and IL-17) by ELISA. CD4^+ ^T cells from immunized mice were co-cultured with CD11c^+ ^cells in the presence of HER2 peptide pool 5 or HIV gag peptide mix for 48 hours. As shown in Figure 2C, in mice immunized with DEC-HER2, we detected a dominant Th1 response with little Th2 or Th17 cytokine secretion from mice immunized with DEC-HER2. T cells from DEC-HER2 immunized mice produced significantly higher amounts of IFNγ but similar levels of IL-4/IL-10/IL-17 in comparison with T cells from mice immunized with Ctrl Ig-HER2 mAb. These data demonstrate that targeting DCs via DEC primed strong Th1 responses against HER2.

### Poly IC induces strong and broad CD4^+ ^T-cell responses to DEC-targeted HER2

**Figure 3:**
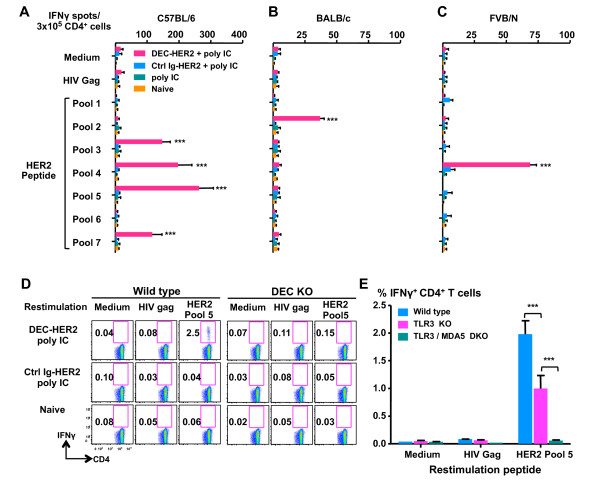
**Poly IC as a mono-adjuvant for strong and broad HER2-specific CD4^+ ^T-cell responses**. C57BL/6 **(A)**, BALB/c **(B)**, or FVB/N **(C) **mice were primed with 5 μg of DEC-HER2 + 50 μg of poly IC and boosted 4 weeks later. Two weeks after the boost, HER2-specific IFNγ production was quantified by enzyme-linked immunosorbent spot assay. All experiments were performed with at least three mice per group, and the results of one of two or three independent experiments are shown. **(D) **Requirement for DEC expression. DEC^-/- ^or wild-type C57BL/6 mice were primed and boosted 4 weeks apart with DEC-HER2 + poly IC, Ctrl Ig-HER2 + poly IC, or nothing. Two weeks after the boost, splenocytes were harvested and re-stimulated with medium alone or 2 μg/mL HIV gag or HER2 peptide pool 5 for 6 hours. IFNγ production was measured by intracellular cytokine staining. Fluorescence-activated cell sorting blots are shown. Three or four mice were in each group, and the results of one of two experiments are shown. **(E) **Requirement for pattern recognition receptors. Wild-type, TLR3 KO, or TLR3/MDA5 DKO C57BL/6 mice were immunized with DEC-HER2 + poly IC. Two weeks after the boost vaccination, splenocytes were harvested and re-stimulated with medium alone or 2 μg/mL HIV gag or HER2 peptide pool 5 for 6 hours. IFNγ production was measured by intracellular cytokine staining. Three mice were in each group. ***P < 0.001. HER, human epidermal growth factor receptor; IFNγ, interferon-gamma; Ig, immunoglobulin; poly IC, polyinosinic/polycytidylic acid; TLR, Toll-like receptor.

As a monotherapy, poly IC has been tested at high doses in patients with cancer and has been shown to have a favorable safety profile [37-41]. To assess the adjuvant activity of poly IC, we primed and boosted mice with DEC-HER2 in combination with 50 μg of poly IC but without anti-CD40 mAb. We found that poly IC as the only adjuvant mediated significant CD4^+ ^T-cell responses in mice immunized with DEC-HER2 mAb but not in mice treated with Ctrl Ig-HER2. We identified four reactive HER2 peptide pools, namely pools 3, 4, 5, and 7 (Figure 3A), which are the same immunogenic pools when poly IC and CD40 Ab were used together as the adjuvants. The mean proportion of CD4^+ ^T cells producing IFNγ/IL-2/TNFα was approximately 50% (right panel of Figure S3 of Additional file 3), which is similar to the response when poly IC and CD40 Ab were co-administrated. Thus, DEC-HER2 vaccination with poly IC can elicit multi-functional CD4^+ ^T-cell responses.

To our knowledge, HER2-specific CD4 T-cell epitopes have not been identified in C57BL/6 mice. To identify the individual reactive CD4 epitopes, we re-stimulated immune CD4^+ ^T cells with single peptides from pools 3, 4, 5, and 7. Seven peptides were able to induce significant IFNγ production by CD4^+ ^T cells from DEC-HER2 immunized mice (Figure S4 of Additional file 4 and Table 1). The dominant CD4 T-cell epitopes in C57BL/6 mice locate within the HER2_284-302 _region (NPEGRYTFGASCVTACPYN).

**Table 1:** Identification of CD4^+ ^T-cell responding peptides in C57BL/6 mice

Peptide pool	Responding 15-mer peptide	Position	Sequence	IFNγ spots per 3 × 10^5 ^CD4^+ ^T cells
3	p72	284-298	NPEGRYTFGASCVTA	206 ± 20.8
4	p73	288-302	RYTFGASCVTACPYN	503 ± 183.5
5	p104	405-423	EITGYLYISAWPDSL	34 ± 7.5
	p105	409-427	YLYISAWPDSLPDLS	27 ± 13.6
	p108	421-435	DLSVFQNLQVIRGRI	25 ± 7.5
	p109	425-438	FQNLQVIRGRILHN	24 ± 7.3
7	p148	571-585	NGSVTCFGPEADQCV	43 ± 12.3

IFNγ, interferon-gamma.

To determine whether DEC-HER2 can induce CD4^+ ^T-cell responses in different MHC backgrounds, we immunized BALB/c (H-2^d^) and FVB/N (H-2^q^) mice. Significant CD4^+ ^T-cell response was induced with DEC-HER2 was targeted to the DEC receptor, although the magnitude was lower than that observed in C57BL/6 mice (Figure 3B, C). The dominant epitope or epitopes presented in BALB/c mice were located in pool 2 (Figure 3B). The amino acid sequence spanning HER2 peptide pool 2 is shown in Table S1 of Additional file 5, and the predicted I-A^d^-restricted epitopes are shown in Table S2 of Additional file 6. CD4^+ ^T-cell responses can also be induced in the FVB/N mice, and the dominant epitope or epitopes are located in HER2 peptide pool 4 (Table S1 of Additional file 5 and Figure 3C).

To determine whether the DEC receptor is essential for vaccination, we immunized DEC^-/- ^mice. DEC-HER2 did not induce HER2-specific T-cell responses in DEC^-/- ^mice (Figure 3D). The dependence of DEC expression in this model is consistent with our previous studies using other immune antigens [32,42].

To determine the pattern recognition receptor required for poly IC, we tested TLR3^-/- ^and TLR3^-/-^MDA5^-/- ^mice. Though significantly reduced, half of the adjuvant effect of poly IC remained intact in TLR3^-/- ^mice. In contrast, poly IC completely lost its adjuvant effect in TLR3^-/-^MDA5^-/- ^mice (Figure 3E). These data suggest that both endosomal TLR3 and the cytosolic sensor MDA5 are required for the maximal adjuvant effect of poly IC.

Thus, targeting HER2 to DCs activated by poly IC induced potent, broad, and multi-functional CD4^+ ^T-cell immunity in mice; these are important features of a successful vaccination. Induction of T-cell immunity was dependent on the expression of DEC receptor for antigen uptake and TLR3/MDA5 for DC maturation.

### HER2 protein targeted to dendritic cells can cross-prime CD8^+ ^T cells

**Figure 4:**
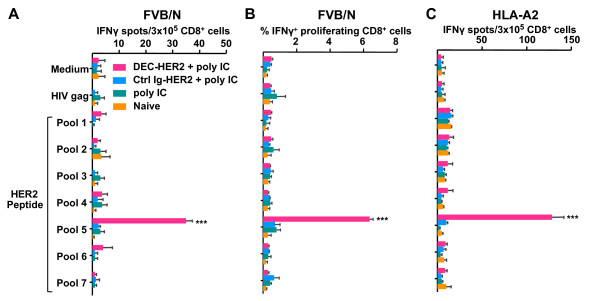
**Cross-presentation of HER2 protein by DEC-HER2 immunization**. **(A) **FVB/N mice were primed and boosted with DEC-HER2 or Ctrl Ig-HER2 (5 μg) in combination with poly IC (50 μg). Two weeks after the boost, spleen CD8^+ ^T cells were purified by magnetic-activated cell sorting and re-stimulated with spleen CD11c^+ ^DCs in the presence of medium alone or 1 μg/mL HIV gag peptide or HER2 peptide pools. IFNγ production was measured by enzyme-linked immunosorbent spot (ELISPOT) assay. **(B) **Proliferative capacity of HER2-specific CD8^+ ^T cells. Mice were immunized as in (A), and bulk splenocytes were labeled with CFSE and re-stimulated with medium or 200 ng/mL HIV gag or HER2 peptide pool 1-7 for 4 days. Cells were re-stimulated for the last 6 hours, and IFNγ production was measured by intracellular cytokine staining. **(C) **HLA-A2 transgenic mice were vaccinated as in (A), and HER2-specific CD8^+ ^T-cell responses were measured by ELISPOT assay. Purified CD8^+ ^T cells were re-stimulated with medium alone or 1 μg/mL HIV gag peptide mix or HER2 peptide pool 1-7. All experiments were performed with at least three mice per group, and the results of one of three experiments are shown. ***P < 0.001. CFSE, 5,6-carboxy fluorescein diacetate succinimidyl ester; HER, human epidermal growth factor receptor; IFNγ, interferon-gamma; Ig, immunoglobulin; poly IC, polyinosinic/polycytidylic acid.

To assess cross-priming of HER2 protein by DEC targeting, we vaccinated FVB/N (H-2^q^) mice with DEC-HER2 or Ctrl Ig-HER2 in combination with poly IC. We found that HER2-specific CD8^+ ^T-cell responses were elicited in mice vaccinated with DEC-HER2 but not in mice vaccinated with Ctrl Ig-HER2 mAb (Figure 4A). The dominant epitope or epitopes are located in peptide pool 5 (Figure 4A), and the amino acid sequence spanning pool 5 is shown in Table S1 of Additional file 5. We also found that HER2-specific CD8^+ ^T cells can proliferate and produce IFNγ when re-stimulated with HER2 peptides (200 ng/mL) (Figure 4B).

To test whether DEC targeting can enhance cross-priming in a human HLA haplotype, we administered DEC-HER2 + poly IC to HLA-A2 transgenic mice on the C57BL/6 background. Although we did not detect cross-priming in wild-type C57BL/6 mice, the HLA-A2 transgenic mice developed HER2-specific CD8^+ ^T-cell responses, as analyzed by ELISPOT assay (Figure 4C). We identified that peptide pool 5 contained the reactive CD8 epitope or epitopes. The amino acid sequence spanning HER2 pool 5 is shown in Table S1 of Additional file 5. Two previously identified HLA-A2-restricted epitopes are located in peptide pool 5. They are HER2_435-443 _(ILHNGAYSL) and HER2_466-474 _(ALIHHNTHL) [43,44]. Thus, targeting HER2 to DCs enhanced not only Th1 responses but also cross-presentation to CD8^+ ^T cells.

### DEC-targeted HER2 vaccination xeno-primes neu-specific CD4^+ ^T cells

**Figure 5:**
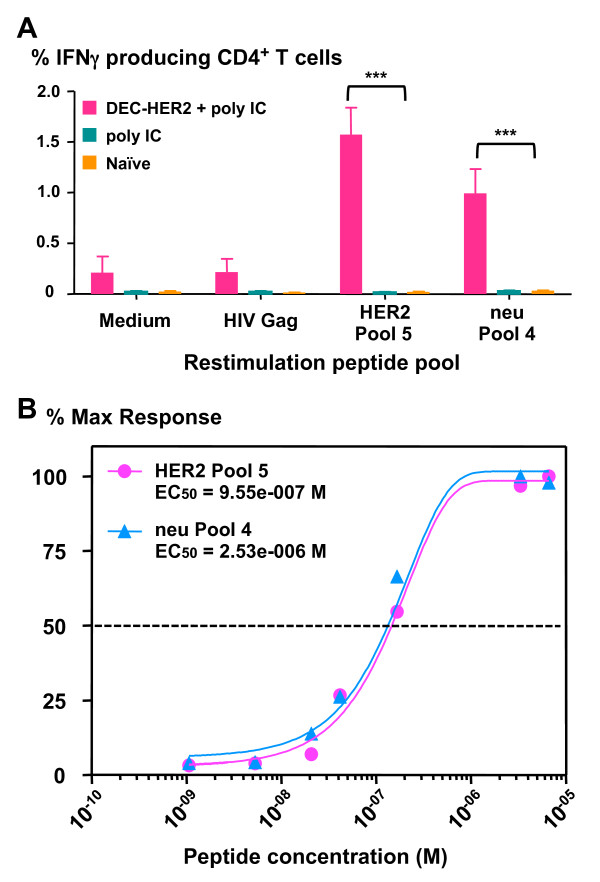
**HER2 immunization primes strong neu-specific CD4^+ ^T-cell responses in mice**. **(A) **C57BL/6 mice were primed with DEC-HER2 in combination with poly IC. Two weeks after the boost, splenocytes were re-stimulated with medium alone, HIV gag peptides, HER2 peptide pool 5, or corresponding neu peptide pool 4 (2 μg/mL). IFNγ production was measured by intracellular cytokine staining. **(B) **Functional avidity of HER2/neu-specific CD4^+ ^T cells. Titrated doses of HER2 or neu peptide pool were used to re-stimulate splenocytes, and IFNγ production was quantified. Data depict the percentage of maximum response at each concentration. ***P < 0.001. Results of two experiments are shown, and six mice were in each group. EC_50_, concentration of peptide that leads to 50% of the maximal responses; HER, human epidermal growth factor receptor; IFNγ, interferon-gamma; poly IC, polyinosinic/polycytidylic acid.

To determine whether T cells induced by human HER2 protein vaccination are able to cross-react with the homologous rat neu protein, we re-stimulated T cells with neu peptides. Despite amino acid sequence differences between human HER2 and rat neu, we found that CD4^+ ^T cells induced by DEC-HER2 vaccination were able to secrete IFNγ upon re-stimulation with the neu peptide pool (Figure 5A). To estimate the functional avidity of the vaccine-induced CD4^+ ^T cells, we re-stimulated splenocytes from mice immunized with DEC-HER2 + poly IC with graded concentrations of HER2 peptide pool 5 or neu peptide pool 4 (from 10 to 0.0016 μg/mL or 6.67 to 0.001 μM). As shown in Figure 5B, HER2- and neu-specific T cells have a similar EC_50 _(that is, concentration of peptide that leads to 50% of the maximal responses). Thus, xeno-priming with HER2 protein can induce homologous rat neu-specific CD4^+ ^T-cell responses; this induction is important for tumor vaccination studies using rat neu-expressing tumor models.

### DEC targeting leads to HER2/neu-specific humoral immunity

**Figure 6:**
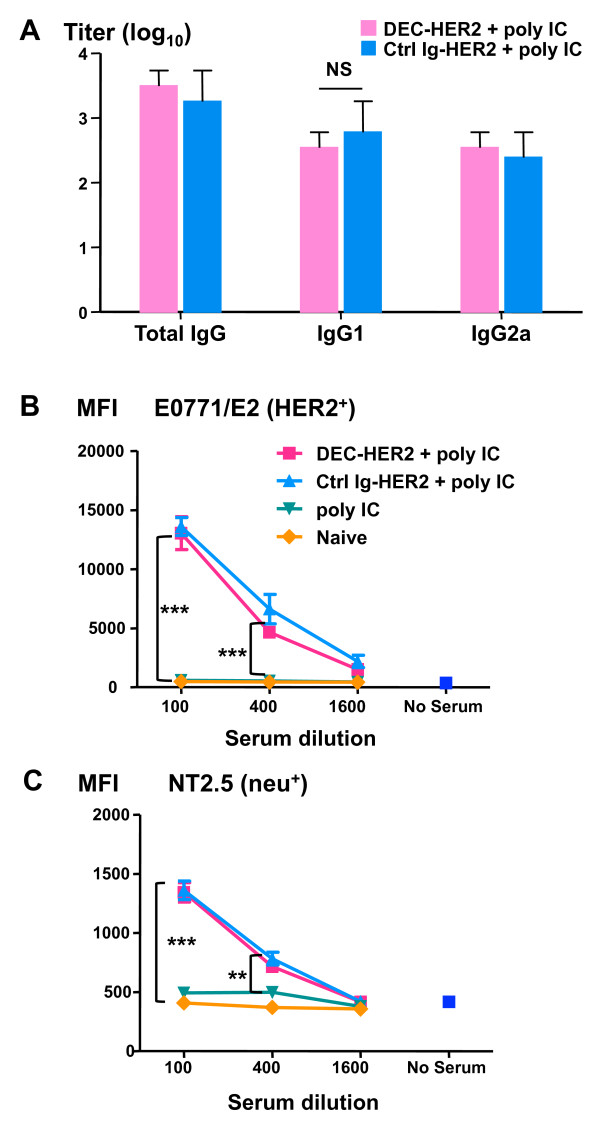
**Induction of anti-HER2/neu humoral immunity**. FVB/N mice were vaccinated with DEC-HER2 or Ctrl Ig-HER2 with poly IC as the adjuvant. Sera were collected 2 weeks after the boost. **(A) **Titers of anti-HER2 IgG were quantified by enzyme-linked immunosorbent assay as described in Materials and methods. **(B, C) **Binding of HER2/neu-expressing tumor cells with immune sera measured by flow cytometry. Sera were diluted 1:100, 1:400, or 1:1,600 and incubated with HER2-expressing E0771/E2 (B) or rat neu-expressing NT2.5 (C) tumor cells for 15 minutes at 4°C. Binding of antibody to tumor cells was detected by staining with anti-mouse IgG-PE antibody and fluorescence-activated cell sorting analysis. Mean fluorescence indices (MFIs) are shown. **P < 0.01, ***P < 0.001. HER, human epidermal growth factor receptor; Ig, immunoglobulin; NS, not statistically significant; PE, phycoerythrin; poly IC, polyinosinic/polycytidylic acid.

To determine whether targeting HER2 to DEC can generate humoral responses, we measured serum anti-HER2 total IgG by ELISA. In FVB/N mice, both DEC-HER2 and Ctrl Ig-HER2 mAbs induced HER2-specific Ab responses with a similar titer of at least 1,800 in total IgG and at least 600 in IgG1 or IgG2a (Figure 6A).

To investigate whether the serum IgG can recognize natural HER2/neu protein presented on tumor cells, we performed flow cytometry-based assays. Serum IgG bound to the HER2-expressing tumor cell line E0771/E2 up to a 1:400 dilution (Figure 6B). Immune sera also bound to neu protein on the surface of NT2.5 tumor cells at a 1:100 dilution, although the binding was much weaker (approximately 10-fold) than binding to HER2 protein (Figure 6C). These data indicate that cross-reactivity of serum HER2-specific IgG to neu protein is weak, unlike what we observed with T-cell cross-reactivity (Figure 5). The baseline mean fluorescence index without immune serum was similar between the two tumor cell lines.

### DEC-HER2 vaccination significantly delays tumor growth

**Figure 7:**
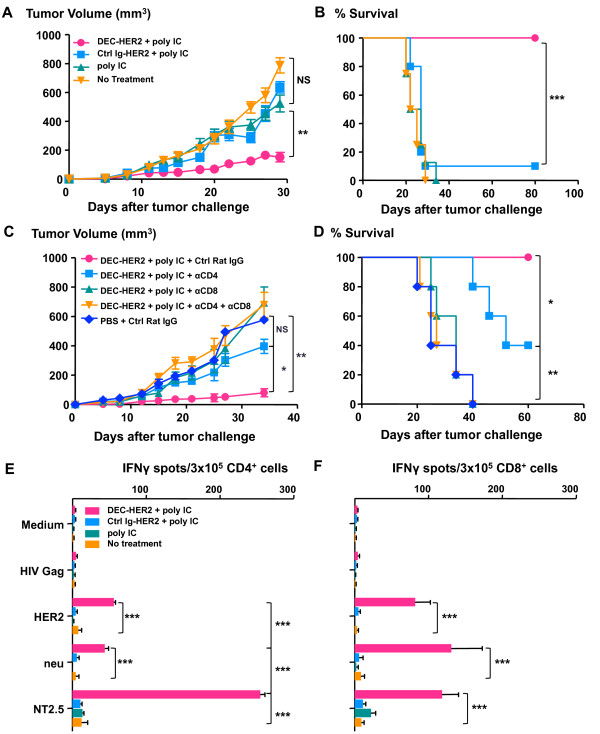
**Targeting HER2 protein to dendritic cells protects mice from a challenge with HER2/neu-expressing mammary tumor cells**. **(A, B) **FVB/N mice were immunized with DEC-HER2 or Ctrl Ig-HER2 with poly IC or poly IC alone or were left untreated. Mice were challenged with one million NT2.5 tumor cells in the mammary fat tissue 10 days after the boost immunization. Tumor growth was monitored by caliper measurement three times a week. Tumor volume (in cubic millimeters) is shown in (A). Survival analysis is shown in (B). Results of three independent experiments (*n *= 5 per group) are shown. **(C, D) **To examine the mechanism of tumor protection, CD4^+^, CD8^+^, or both types of T cells were depleted after immunization (before tumor challenge) as described in Materials and methods. Mice were challenged with one million NT2.5 tumor cells, and tumor growth curves are shown in (C). Survival analysis is shown in (D). Results of two independent experiments (*n *= 10 mice per group) are shown. **(E, F) **Anti-tumor immunity was determined 14 days after tumor challenge. Splenic CD4^+ ^and CD8^+ ^cells were purified by magnetic-activated cell sorting isolation. Splenic CD11c^+ ^cells were purified from naïve FVB/N mice. CD4^+ ^(E) or CD8^+ ^(F) T cells were re-stimulated with CD11c^+ ^cells pulsed with 1 μg/mL of HIV gag, HER2 peptide pool 5, or neu peptide pool 4 or 10 μg/mL NT2.5 tumor lysate at a T cell/DC ratio of 3:1 for 3 days. IFNγ production was measured by enzyme-linked immunosorbent spot assay. Four mice were in each group; the results of one of two independent experiments are shown. *P < 0.05, **P < 0.01, ***P < 0.001. HER, human epidermal growth factor receptor; IFNγ, interferon-gamma; Ig, immunoglobulin; NS, not statistically significant; PBS, phosphate-buffered saline; poly IC, polyinosinic/polycytidylic acid.

To assess whether the vaccine-induced HER2/neu-specific T-cell immunity can mediate protective anti-tumor immunity, we immunized FVB/N mice with DEC-HER2 or Ctrl Ig-HER2 in combination with poly IC. Ten days after boost immunization, mice were challenged with NT2.5 tumor cells in the mammary fat tissue. Vaccination with only 5 μg of DEC-HER2 protein with poly IC significantly delayed tumor growth (Figure 7A). Although Ctrl Ig-HER2 vaccination was able to induce anti-HER2 Ab responses (Figure 6), it was unable to protect mice from tumor outgrowth. As expected, administration of poly IC alone did not delay tumor growth. Although not all of the mice that received DEC-HER2 + poly IC vaccinations were tumor-free, the survival rate (up to 80 days) of DEC-HER2-immunized mice was significantly greater than that of Ctrl Ig-HER2-treated or untreated mice.

To determine the underlying tumor protection mechanism, we depleted CD4^+^, CD8^+^, or both populations at the effector phase, the time of tumor challenge. Depletion efficiency was verified by FACS analysis of peripheral blood lymphocytes the day before tumor challenge (Figure S5 of Additional file 7). We found that both CD4^+ ^and CD8^+ ^T cells were required for tumor protection in the vaccinated mice as determined by tumor growth kinetics (Figure 7C). Survival analysis (up to 60 days) shows that depletion of CD4^+ ^T cells did not completely abrogate survival benefit of DEC-HER2 vaccination. However, depletion of CD8^+ ^T cells completely abrogated the tumor protection effect (Figure 7D). These data suggest that CD8^+ ^T cells play a more critical role for long-term survival than CD4^+ ^T cells do.

To determine the induction of anti-tumor T-cell responses, we harvested the splenocytes 14 days after tumor challenge and re-stimulated purified CD4^+ ^or CD8^+ ^T cells with purified splenic CD11c^+ ^cells pulsed with peptides mix (HIV gag, HER2, or neu) or NT2.5 tumor lysate. Strong HER2 and neu-specific CD4^+ ^(Figure 7E) and CD8^+ ^(Figure 7F) T-cell responses against HER2/neu were induced by DEC-HER2 but not by Ctrl Ig-HER2. CD4^+ ^and CD8^+ ^T cells also produced IFNγ upon re-stimulation with NT2.5 tumor lysate-pulsed DCs. Thus, DEC-HER2 xeno-priming significantly increased anti-tumor immunity and this increase led to a significant delay in tumor outgrowth. Thus, vaccine-induced CD4^+ ^and CD8^+ ^T cells were both essential for tumor protection and long-term survival.

## Discussion

In this study, we found that delivery of the HER2 tumor antigen within DEC mAb allowed efficient immunization of HER2/neu-specific T cells at a low dose (5 μg of chimeric Ab or 2.7 μg of HER2 protein). We also demonstrated that vaccine-induced T-cell immunity significantly delayed neu-expressing tumor growth in mice.

To overcome the weak T-cell immunity that is typically elicited by HER2 protein vaccines, we delivered HER2 to the DEC^+ ^DCs *in vivo*. High efficiency of targeting tumor antigen to DEC^+ ^DCs allows a significantly lower dose of protein to achieve potent CD4^+ ^and CD8^+ ^T-cell responses. We found that only a single dose of 5 μg of chimeric mAb (equivalent to 2.7 μg of HER2 protein) was able to induce strong CD4^+ ^T-cell immunity in mice (Figure 2). On the other hand, at the same dose, linking HER2 protein to an isotype control mAb was inefficient in inducing T-cell immunity. Similarly, studies from other groups have shown that usually a much higher dose of soluble HER2/neu protein is required to induce detectable T-cell immunity [13-15,17,45]. Even at two doses of 50 μg, soluble HER2 protein induces only marginal T-cell immunity [14]. The robust CD4^+ ^T-cell responses induced by vaccination are Th1-dominant, as measured by high IFNγ but low IL-4/IL-10/IL-17 production (Figure 2C). Increased Th1 immunity usually is associated with a better outcome in patients with cancer [46,47].

The efficient induction of CD4^+ ^T-cell responses by our vaccine approach is dependent on the expression of DEC receptor. In DEC^-/- ^mice, we did not detect significant HER2-specific T-cell immunity (Figure 3D). This result is consistent with our previous vaccination studies using other antigens [32,42]. The T-cell tolerance induced by steady-state immature DCs was overcome by administration of TLR3 agonist poly IC with the protein vaccine. We demonstrated that recognition of poly IC by its cellular receptors TLR3 and MDA5 is essential for the induction of HER2 immunity (Figure 3E) and this is consistent with our previous findings [48]. We observed a slightly increased background response to HER2 when poly IC was combined with DEC-HER2 vaccination, but the background response significantly decreased at later time points (> 3 weeks) after immunization (unpublished results). The observed temporary general immune stimulation is most likely due to the bystander T-cell activation caused by the high amounts of IFNγ, IL-2, and TNFα secreted by the vaccine-induced T cells (Figure S3 of Additional file 3). These activated bystander T cells might in turn amplify vaccine-induced immune response against the tumor antigen and this would represent a beneficial outcome of our vaccine strategy.

Targeting HER2 to activated DCs enhanced not only the magnitude but also the quality of the CD4^+ ^T-cell responses in four ways. First, broad T-cell responses were developed in three MHC haplotypes (H-2^d^, H-2^b^, and H-2^q^) tested here (Figure 3A and Table 1). Second, the vaccine-induced T cells produced multiple cytokines (IFNγ, TNFα, and IL-2), which are important in regulating the expansion of CD4^+ ^and CD8^+ ^T cells (Figure S3 of Additional file 3). Third, the vaccine-induced HER2-specific CD4^+ ^T cells proliferated rigorously and secreted IFNγ upon antigen challenge (Figure 2B). Fourth, the HER2-specific CD4^+ ^T cells cross-reacted to rat neu antigen with similar functional avidity (Figure 5), despite the sequence differences between the two homologs.

The high quantity and quality of vaccine-induced CD4^+ ^T-cell responses have several implications for tumor immunotherapy. First, they can enhance the magnitude and longevity of CD8^+ ^T-cell immunity and promote infiltration of CD8^+ ^T cells into the tumor milieu [49-51]. This is supported by vaccine studies reported by Knutson and colleagues [52], who studied patients with breast cancer. The clinical data of the authors indicate that immunization with a peptide vaccine designed to stimulate CD8^+ ^T cells alone generates only low and short-lived immune responses [52]. Second, HER2/neu-specific Th1 cells can home to the tumor site, secrete IFNγ and other inflammatory cytokines in the tumor microenvironment, and boost the function of macrophages and DCs [53,54]. Activation of APCs may increase processing and presentation of endogenous tumor antigens from dying cells, resulting in 'epitope spreading', which refers to the development of immunity to tumor antigens other than HER2/neu and which could halt the progression of HER2/neu-negative variants [55]. Third, CD4^+ ^T cells are also cytotoxic directly against tumor cells [56-58], although the tumor cells that we evaluated here lack MHC II (unpublished results).

Immunotherapy approaches that induce integrated CD4^+ ^and CD8^+ ^T-cell responses are desirable. Here, we show that DEC-HER2 induced not only strong CD4^+ ^T-cell responses but also significant CD8^+ ^T-cell responses at a low dose (Figure 4). These CD8^+ ^T cells proliferate rigorously upon re-stimulation with HER2 peptide *in vitro *(Figure 4B). Importantly, using HLA-A2 transgenic mice, we found that CD8^+ ^T-cell responses can be induced in different MHC haplotypes (Figure 4C). The responding HER2 peptide pool 5 indeed contains two A2-restricted HER2 epitopes that have been described previously [43,44]. This result is consistent with previous findings that targeting protein to activated DCs, especially CD8α^+ ^DCs, can significantly enhance antigen cross-presentation to CD8^+ ^T cells [27,31,59,60].

Targeting HER2 to DEC^+ ^DCs induced not only integrated CD4^+ ^and CD8^+ ^T-cell responses but also serum Ab response (Figure 6). Importantly, HER2/neu-specific IgG induced by immunization can recognize naturally derived HER2/neu epitopes that are expressed on HER2/neu-expressing tumor cells (Figure 6B, C). Although HER2-specific Ab responses between DC-targeted or non-targeted HER2 protein were similar as assessed by titer and isotypes, the breadth and functional qualities of the Ab could be different.

Strong HER2-specific immunity induced by DEC-targeting immunization is translated into significant anti-tumor responses in a transplantable tumor model in FVB/N mice. Xeno-priming mice with 5 μg of DEC-HER2 protein in combination with poly IC significantly delayed the development of transplantable neu-expressing tumor (Figure 7). Vaccination not only delayed the growth of the tumors (Figure 7A) but also improved the long-term overall survival of the mice (Figure 7B). These results indicate that an integrated CD4^+ ^and CD8^+ ^T-cell immunity is the major tumor protection mechanism in neu-expressing tumor-challenged FVB/N mice (Figure 7C, D). These results are consistent with a previous report [61].

We found that CD8^+ ^T cells are playing a more dominant role in tumor protection as depletion of CD8^+ ^T cells had a more dramatic effect on tumor growth and overall survival. Induction of HER2/neu-specific T-cell immunity was confirmed by *in vitro *T-cell assays (Figure 7E, B). Interestingly, we observed a robust CD4^+ ^T-cell response when neu-expressing tumor-lysate-pulsed DCs were used as the antigen source. The response is even stronger than that using HER2/neu-peptide-pulsed DCs as stimuli. There are three possible explanations for this unexpected finding. First, the quantity of neu epitopes presented on DCs may be higher with tumor-lysate-pulsed DCs. Second, epitope spreading could be induced with DEC-HER2 vaccination. Therefore, the higher responses could represent a cumulative response against HER2/neu and other tumor antigens. Third, post-translational modifications (glycosylation and phosphorylation and so on) presented on naturally derived neu epitopes, but not on synthetic peptides, may boost T-cell recognition and enhance TCR signal strength, resulting in a stronger CD4^+ ^T-cell activation. In summary, our results show that targeting HER2 protein to activated DCs *in situ *significantly enhances anti-tumor T-cell immunity, and we propose that this strategy provides a feasible approach for immunotherapy in patients with cancer.

## Conclusions

We demonstrated that immunization of mice with a HER2 protein vaccine targeting DEC^+ ^DCs *in vivo *induced high levels of T- and B-cell responses as determined by various *in vitro *immune assays. Non-targeted HER2 protein was poorly immunogenic for CD4^+ ^and CD8^+ ^T cells. Analysis of the quality of vaccine-induced Th1 cells revealed multiple features that favor anti-tumor immunity, such as breadth, proliferative capacity, and multiple cytokine production. This vaccination approach protected mice from neu-expressing tumor outgrowth following as little as 2.7 μg of HER2 protein in the vaccine. Vaccine-induced CD4^+ ^and CD8^+ ^T cells were both essential for tumor protection. This vaccine approach is feasible and cost-efficient in clinical settings. Since a fully humanized DEC mAb has been brought into phase I clinical trials with HIV antigens, this vaccine strategy would seem logical to pursue in the development of vaccines for patients with breast cancer.

## Abbreviations

Ab: antibody; Ag: antigen; APC: antigen-presenting cell; BSA: bovine serum albumin; CFSE: 5,6-carboxy fluorescein diacetate succinimidyl ester; CHO: Chinese hamster ovary; DC: dendritic cell; ELISA: enzyme-linked immunosorbent assay; ELISPOT: enzyme-linked immunosorbent spot; FACS: fluorescence-activated cell sorting; HER: human epidermal growth factor receptor; HRP: horseradish peroxidase; IFNγ: interferon-gamma; Ig: immunoglobulin; IL: interleukin; mAb: monoclonal antibody; MACS: magnetic-activated cell sorting; MHC: major histocompatibility complex; PBS: phosphate-buffered saline; PE: phycoerythrin; poly IC: polyinosinic/polycytidylic acid; poly ICLC: polyinosinic:polycytidylic acid stabilized with poly-L-lysine; Th: T helper; TLR: Toll-like receptor; TNF: tumor necrosis factor.

## Competing interests

RMS had financial interests in Celldex Therapeutics, Inc., which is developing DEC antibodies for human use. L-ZH and TK are current employees of Celldex Therapeutics, Inc. The other authors declare that they have no competing interests.

## Authors' contributions

The contributions of the authors - with the exception of RMS, who supervised all aspects of this research and the preparation of this manuscript - are reflected in the order shown. BW performed most of the immune assays, animal experiments, and laboratory analysis and wrote the original manuscript. NZ performed flow cytometry assays and animal experiments and helped draft and edit the manuscript. L-ZH carried out molecular cloning and prepared HER2 soluble protein. LZ was responsible for fusion antibody production, animal tumor experiments, and mouse colony maintenance. JMYK was responsible for hybridoma cell culture and mAb purification. TK participated in data interpretation and was involved in drafting, critically reviewing, and revising the manuscript. All authors read and approved the final manuscript.

## Supplementary Material

Additional file 1 **Figure S1**. Characterization of FLAG-tagged HER2 recombinant protein. Extracellular domain of HER2 was cloned into FLAG-His-tagged expression vector (pS:FLAG-His). FLAG-HER2 protein was produced by transient transfection of 293T cells and further purified with anti-FLAG column (Sigma). The quality of FLAG-HER2 protein was checked with SDS-PAGE gel under non-reducing condition. 2 μg protein was loaded in indicated well. Click here for file

Additional file 2 **Figure S2**. Effect of agonistic CD28 mAb on intracellular cytokine staining assay. (**A**) C57BL/6 mice were immunized with DEC-HER2+poly IC. Two weeks after boost, splenocytes were restimulated with medium alone, HIV gag peptides, or HER2 peptide pool 1-7 with or without CD28 mAb (2 μg/mL) during the 6 h simulation. IFNγ production was measured by intracellular cytokine staining. (**B**) Functional avidity of CD4^+ ^T cells. Mice were immunized as in (**A**), splenocytes were restimulated with titrated dose of HER2 peptide pool 4 and IFNγ production was measured by intracellular cytokine staining. Data depicts the percentage of maximum response at each concentration. Click here for file

Additional file 3 **Figure S3**. Functional characterization of IL-2-, IFNγ- or TNFα-producing CD4^+ ^T cells by multiparameter flow cytometry. C57BL/6 mice were immunized with DEC-HER2+poly IC. Two weeks after boost immunization, splenocytes were restimulated with 2 μg/mL of HER2 peptide pool 5 and analyzed for cytokines production by FACS. The pie charts show the quality of the cytokine response, comprised of seven functionally distinct populations producing IL-2, IFNγ- and TNFα, individually or in any combination. The percentages are based on the production of the respective cytokines within the live CD3^+^CD4^+ ^population. Click here for file

Additional file 4 **Figure S4**. Identification of HER2-specific CD4^+ ^T cell epitopes in C57BL/6 mice. Mice were immunized with DEC-HER2+poly IC. Two weeks after the boost immunization, splenic CD4^+ ^and CD11c^+ ^cells were isolated and cocultured in the presence of 2 μg/mL indicated individual HER2 peptide from pool 3, 4, 5, and 7. IFNγ production was quantified by ELISPOT assay. The ID of responding HER2 peptide is indicated above the corresponding bar. Click here for file

Additional file 5 **Table S1**. Immunogenic peptide sequences. Click here for file

Additional file 6 **Table S2**. Prediction of I-A^d ^restricted HER2 epitopes. Click here for file

Additional file 7 **Figure S5**. Depletion efficiency of CD4^+ ^and CD8^+ ^T cells in peripheral blood analyzed by flow cytometry. The day before tumor challenge, peripheral blood cells were harvested by submandibular bleeding and depletion efficiency was analyzed by flow cytometry. Live CD3^+ ^cells were gated for CD4^+^/CD8^+ ^population analysis. Shown FACS dot plot from one representative mouse. Click here for file
