# Supplementary figures and images for: Targeting of the non-mutated tumor antigen HER2/neu to mature dendritic cells induces an integrated immune response that protects against breast cancer in mice
Source: Breast Cancer Res. 2012 Mar 7;14(2):R39. doi: 10.1186/bcr3135 (PMC3446373; doi:10.1186/bcr3135)

Figure S1

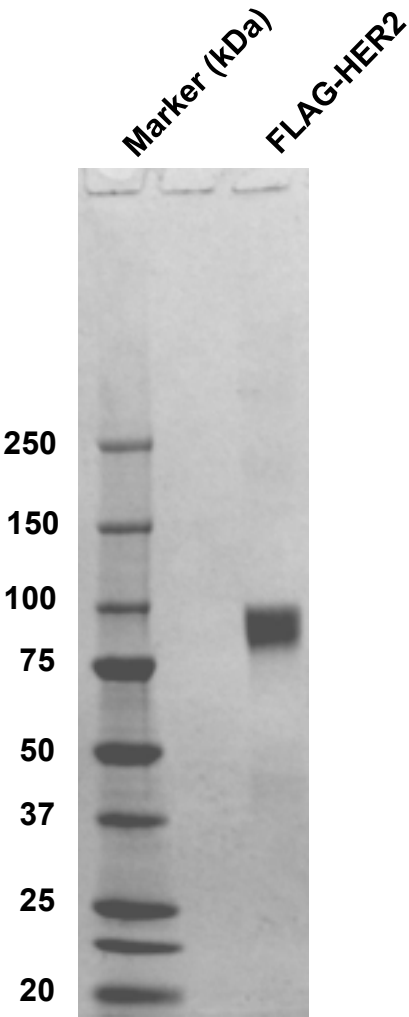

Supplement: Additional file 1 — Figure S1. Characterization of FLAG-tagged HER2 recombinant protein. Extracellular domain of HER2 was cloned into FLAG-His-tagged expression vector (pS:FLAG-His). FLAG-HER2 protein was produced by transient transfection of 293T cells and further purified with anti-FLAG column (Sigma). The quality of FLAG-HER2 protein was checked with SDS-PAGE gel under non-reducing condition. 2 μg protein was loaded in indicated well. [file bcr3135-S1.PDF]

Figure S2

A

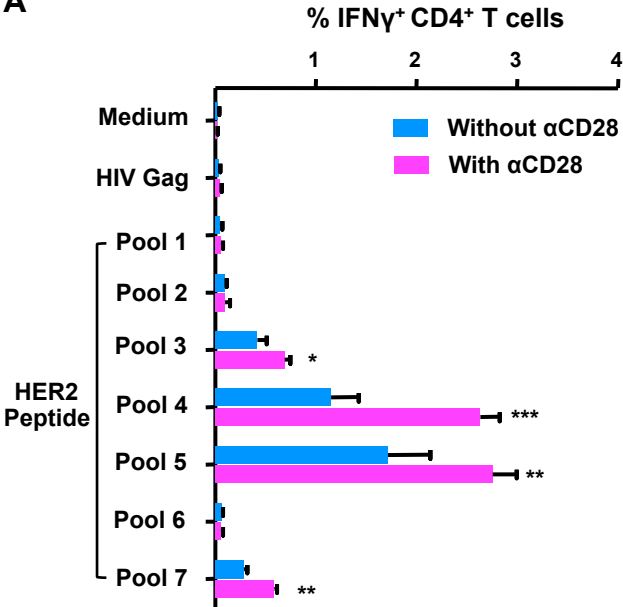

B % Max Response

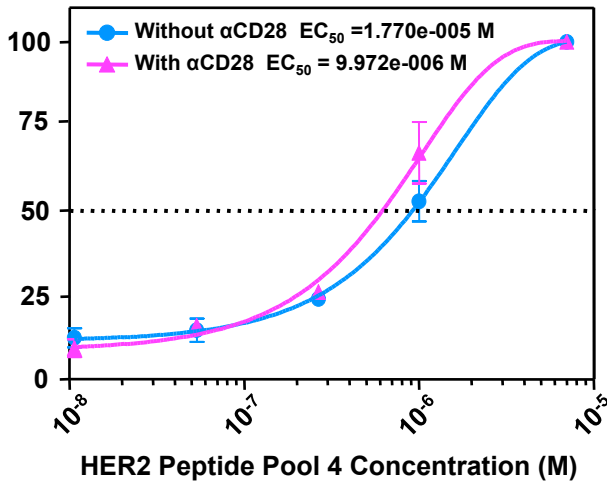

Supplement: Additional file 2 — Figure S2. Effect of agonistic CD28 mAb on intracellular cytokine staining assay. (A) C57BL/6 mice were immunized with DEC-HER2+poly IC. Two weeks after boost, splenocytes were restimulated with medium alone, HIV gag peptides, or HER2 peptide pool 1-7 with or without CD28 mAb (2 μg/mL) during the 6 h simulation. IFNγ production was measured by intracellular cytokine staining. (B) Functional avidity of CD4+ T cells. Mice were immunized as in (A), splenocytes were restimulated with titrated dose of HER2 peptide pool 4 and IFNγ production was measured by intracellular cytokine staining. Data depicts the percentage of maximum response at each concentration. [file bcr3135-S2.PDF]

Figure S3

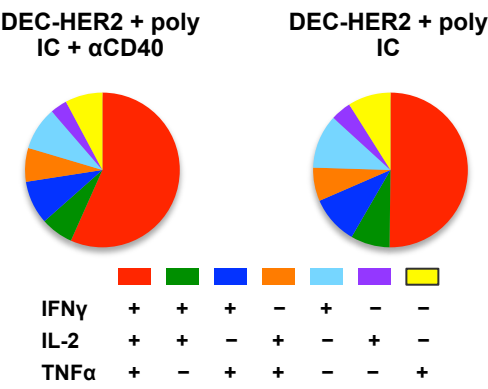

Supplement: Additional file 3 — Figure S3. Functional characterization of IL-2-, IFNγ- or TNFα-producing CD4+ T cells by multiparameter flow cytometry. C57BL/6 mice were immunized with DEC-HER2+poly IC. Two weeks after boost immunization, splenocytes were restimulated with 2 μg/mL of HER2 peptide pool 5 and analyzed for cytokines production by FACS. The pie charts show the quality of the cytokine response, comprised of seven functionally distinct populations producing IL-2, IFNγ- and TNFα, individually or in any combination. The percentages are based on the production of the respective cytokines within the live CD3+CD4+ population. [file bcr3135-S3.PDF]

Figure S4

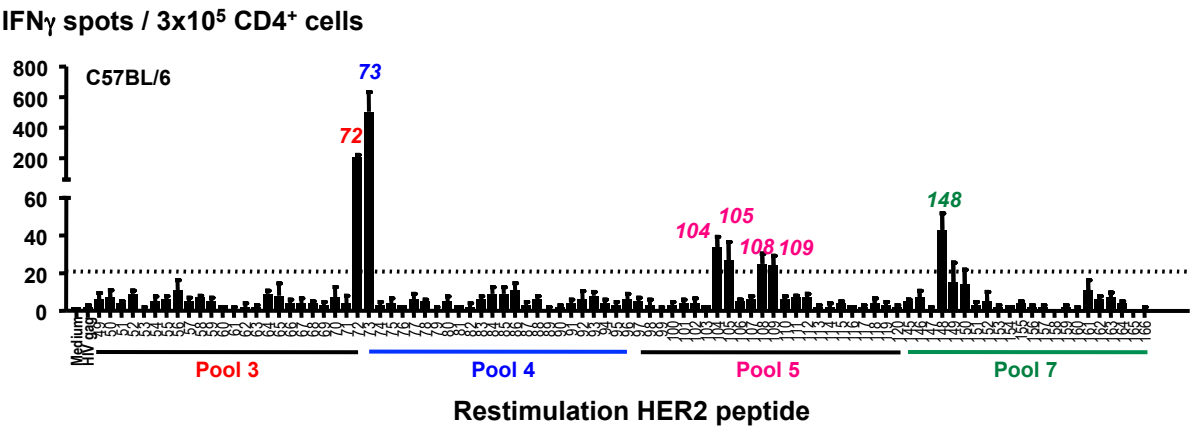

Supplement: Additional file 4 — Figure S4. Identification of HER2-specific CD4+ T cell epitopes in C57BL/6 mice. Mice were immunized with DEC-HER2+poly IC. Two weeks after the boost immunization, splenic CD4+ and CD11c+ cells were isolated and cocultured in the presence of 2 μg/mL indicated individual HER2 peptide from pool 3, 4, 5, and 7. IFNγ production was quantified by ELISPOT assay. The ID of responding HER2 peptide is indicated above the corresponding bar. [file bcr3135-S4.PDF]

Figure S5

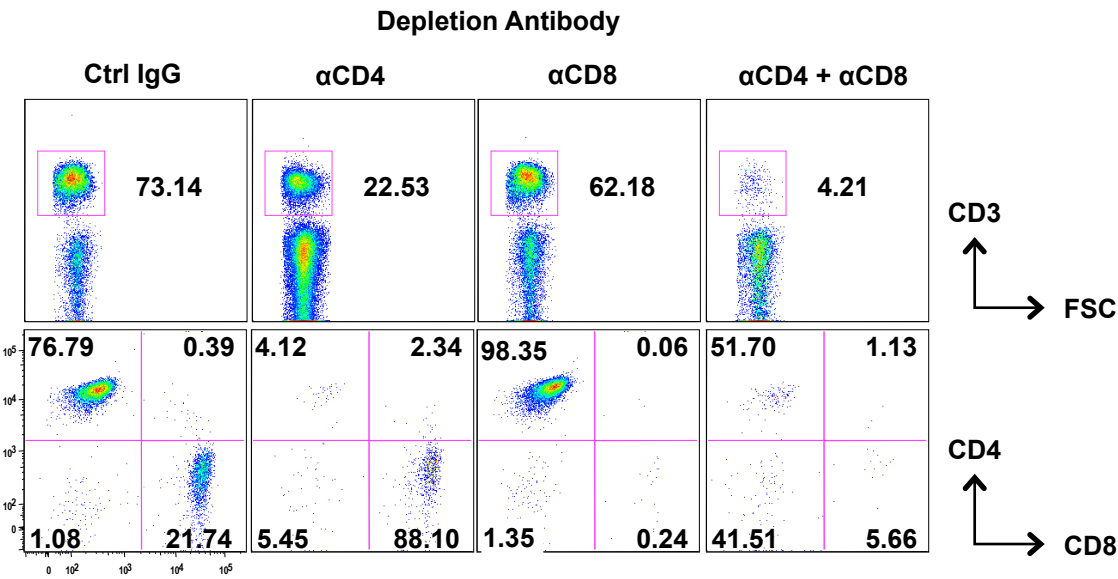

Supplement: Additional file 7 — Figure S5. Depletion efficiency of CD4+ and CD8+ T cells in peripheral blood analyzed by flow cytometry. The day before tumor challenge, peripheral blood cells were harvested by submandibular bleeding and depletion efficiency was analyzed by flow cytometry. Live CD3+ cells were gated for CD4+/CD8+ population analysis. Shown FACS dot plot from one representative mouse. [file bcr3135-S7.PDF]
