# Supplementary material for: Targeting of the non-mutated tumor antigen HER2/neu to mature dendritic cells induces an integrated immune response that protects against breast cancer in mice
Source: Breast Cancer Res. 2012 Mar 7;14(2):R39. doi: 10.1186/bcr3135 (PMC3446373; doi:10.1186/bcr3135)
Supplement: Additional file 5 — Table S1. Immunogenic peptide sequences. [file bcr3135-S5.DOC]

**Table S1: Immunogenic peptide sequences.**

|  | **Strain** | **HER2 Peptide Pool** | **Position** | **Sequence** |
| --- | --- | --- | --- | --- |
| **CD4** | BALB/C | 2 | 111-215 | NYALAVLDNGDPLNNTTPVTGASPGGLRELQLRSLTEILKGGVLIQRNPQLCYQDTILWKDIFHKNNQLALTLIDTNRSRACHPCSPMCKGSRCWGESSEDCQSL |
|  | FVB/N | 4 | 288-388 | RYTFGASCVTACPYNYLSTDVGSCTLVCPLHNQEVTAEDGTQRCEKCSKPCARVCYGLGMEHLREVRAVTSANIQEFAGCKKIFGSLAFLPESFDGDPASN |
| **CD8** | FVB/N  A2 Tg | 5 | 378-477 | PESFDGDPASNTAPLQPEQLQVFETLEEITGYLYISAWPDSLPDLSVFQNLQVIRGRILHNGAYSLTLQGLGISWLGLRSLRELGSGLALIHHNTHLCFVHTV |
