# Supplementary material for: Targeting of the non-mutated tumor antigen HER2/neu to mature dendritic cells induces an integrated immune response that protects against breast cancer in mice
Source: Breast Cancer Res. 2012 Mar 7;14(2):R39. doi: 10.1186/bcr3135 (PMC3446373; doi:10.1186/bcr3135)
Supplement: Additional file 6 — Table S2. Prediction of I-Ad restricted HER2 epitopes. [file bcr3135-S6.DOC]

**Table S2: Prediction of I-A^d^** restricted HER2 epitopes

| **Position** | **Sequence** | **Score** |
| --- | --- | --- |
| 134-148 | PGGLRELQLRSLTEI | 22 |
| 124-138 | NNTTPVTGASPGGLR | 20 |
| 178-192 | QLALTLIDTNRSRAC | 20 |
| 173-187 | FHKNNQLALTLIDTN | 18 |
| 121-135 | DPLNNTTPVTGASPG | 16 |

*Predicted using **SYFPEIHI Database** [[http://www.syfpeithi.de](http://www.syfpeithi.de/)]

| **Position** | **Sequence** | **Core Sequence** | **IC50**  **(nM)** | **Percentile**  **Rank** |
| --- | --- | --- | --- | --- |
| 136-150 | GLRELQLRSLTEILK | RELQLRSLT | 2314 | 1.58 |
| 137-151 | LRELQLRSLTEILKG | LRSLTEILK | 2625 | 2.37 |
| 135-149 | GGLRELQLRSLTEIL | LRELQLRSL | 3972 | 3.15 |
| 134-148 | PGGLRELQLRSLTEI | LRELQLRSL | 4201 | 3.66 |
| 175-189 | KNNQLALTLIDTNRS | QLALTLIDT | 3122 | 3.85 |

*Predicted using **IEDB Analysis Resource** [<http://tools.immuneepitope.org/analyze/html/mhc_binding.html>]
